# Health taxes: a call for papers

**DOI:** 10.1136/bmjgh-2022-010709

**Published:** 2022-10-03

**Authors:** Robert Marten, Jeremias Paul, Tessa Tan Torres Edejer, Diarmid Campbell-Lendrum

**Affiliations:** 1 Alliance for Health Policy and Systems Research, WHO Headquarters, Geneva, Switzerland; 2 Department of Health Promotion, WHO Headquarters, Geneva, Switzerland; 3 Department of Health Systems Governance and Financing (HGF), WHO Headquarters, Geneva, Switzerland; 4 Department of Environment, Climate and Health, WHO Headquarters, Geneva, Switzerland

**Keywords:** health policy, health economics

Health taxes are levies on products that harm human and planetary health.^1^ They are critical policy tools to advance public health; health taxes save and improve millions of lives and generate resources to invest in health and other developmental priorities.^2^ However, health taxes are also one of governments’ most underused interventions. A 2021 WHO report showed that only 13% of the world’s population is covered by best-practice levels of tobacco taxes despite being the single most effective way to reduce tobacco use.^3^ There are important linkages across tobacco, alcohol and sugar-sweetened beverages in terms of how taxes are designed, developed and implemented.^4^ There is also growing interest for health taxes on products like fossil fuels,^5^ meat,^6^ as well as salt among others. Accordingly, governments are requesting WHO’s technical assistance.

Yet emerging evidence suggests how they are framed^7^ and developed within their national political, economic and social context matters^8 9^; indeed, this context shapes implementation and often determines success. For example, health taxes are often successfully portrayed by corporate actors and vested interests as attacks on individual and consumer rights instead of being championed by public health practitioners as reasonable ways to contain corporate interests and protect, reduce deaths and improve human lives.^10^ The strategic consideration and development of health tax policies must be considered in terms of the broader commercial determinants of health as well as within the context of power and politics. A lack of appreciation for political challenges can hamper national adaptation and adoption.^11^ While any health policy process is inherently political, those opposed to health taxes have deep pockets and political influence; applying health policy and political economy analysis can improve policy design and accelerate implementation, saving and improving millions of human lives.^12^

To do this, governments require sustainable, local support to understand and analyse how to best position and frame health taxes politically. Locally generated evidence and policy analysis, with a deep understanding of the contextual politics, processes and powers, are crucial. Although countries face unique challenges in implementing programmes, research that is grounded in local settings and context can help improve design and overcome barriers accelerating implementation. By providing the analysis and evidence to inform civil society advocacy as well as improve government policymaking, health policy analysis can ultimately improve and strengthen how interventions are framed, designed and implemented.

To fill this gap, the Alliance for Health Policy and Systems Research, in collaboration with WHO Departments and the Inter-Agency Working Group on Health Taxes, is supporting the development of a series of analytical country case studies to better understand the political economy of advancing health taxes in eight countries (namely, Bangladesh, Ethiopia, Ghana, Indonesia, Nepal, Pakistan, Peru and Vietnam). To complement this work and advance the field, we are now issuing an open call for papers for a special issue of *BMJ Global Health* on health taxes.

We are interested in papers that focus on political economy and policy analysis as well as consider how framing can be used to advance health taxes; we are interested in health taxes on products including, but not limited to, tobacco, alcohol, sugar, fossil fuels, meat and salt. The special issue welcomes a variety of different types of articles, including those focused on exploring new theoretical and methodological terrain, in addition to papers that present empirical research findings considering how countries can accelerate, develop, deepen, expand and sustain health taxes, with a special interest on low-income and middle-income countries. We call for submissions across article types, including original research, analysis and practice articles by 17 February 2023.

## Data Availability

There are no data in this work.
